# Chronic CSE Treatment Induces the Growth of Normal Oral Keratinocytes via PDK2 Upregulation, Increased Glycolysis and HIF1α Stabilization

**DOI:** 10.1371/journal.pone.0016207

**Published:** 2011-01-19

**Authors:** Wenyue Sun, Steven S. Chang, Yumei Fu, Yan Liu, Joseph A. Califano

**Affiliations:** 1 Department of Otolaryngology-Head and Neck Surgery, Johns Hopkins Medical Institutions, Baltimore, Maryland, United States of America; 2 Department of Surgery, Johns Hopkins Medical Institutions, Baltimore, Maryland, United States of America; 3 Milton J. Dance Head and Neck Center, Greater Baltimore Medical Center, Baltimore, Maryland, United States of America; Yale Medical School, United States of America

## Abstract

**Background:**

Exposure to cigarette smoke is a major risk factor for head and neck squamous cell carcinoma (HNSCC). We have previously established a chronic cigarette smoke extract (CSE)-treated human oral normal keratinocyte model, demonstrating an elevated frequency of mitochondrial mutations in CSE treated cells. Using this model we further characterized the mechanism by which chronic CSE treatment induces increased cellular proliferation.

**Methodology/Principal Findings:**

We demonstrate that chronic CSE treatment upregulates PDK2 expression, decreases PDH activity and thereby increases the glycolytic metabolites pyruvate and lactate. We also found that the chronic CSE treatment enhanced HIF1α accumulation through increased pyruvate and lactate production in a manner selectively reversible by ascorbate. Use of a HIF1α small molecule inhibitor blocked the growth induced by chronic CSE treatment in OKF6 cells. Furthermore, chronic CSE treatment was found to increase ROS (reactive oxygen species) production, and application of the ROS scavengers N-acetylcysteine abrogated the expression of PDK2 and HIF1α. Notably, treatment with dichloroacetate, a PDK2 inhibitor, also decreased the HIF1α expression as well as cell proliferation in chronic CSE treated OKF6 cells.

**Conclusions/Significance:**

Our findings suggest that chronic CSE treatment contribute to cell growth via increased ROS production through mitochondrial mutations, upregulation of PDK2, attenuating PDH activity thereby increasing glycolytic metabolites, resulting in HIF1α stabilization. This study suggests a role for chronic tobacco exposure in the development of aerobic glycolysis and normoxic HIFα activation as a part of HNSCC initiation. These data may provide insights into development of chemopreventive strategies for smoking related cancers.

## Introduction

Cigarette smoke accounts for 30% of all cancer related deaths in the United States. Cigarette smoke has been linked to a variety of malignancies and is an important factor that is causally associated with HNSCC [1]. More than 100 carcinogens, mutagens, and tumor promoters have been identified in tobacco smoke. Recently, the relationship between mitochondrial alterations and cigarette smoke has been investigated. It is known that mitochondrial DNA content increase in response to cigarette smoking, and even several decades after cigarette smoking, mitochondrial DNA content alterations in response to cigarette smoking still persist [2]. Moreover, cigarette smoke exposure affects mitochondrial DNA (mtDNA) mutations in buccal cells of smokers. mtDNA mutation density was significantly higher in smokers than in non-smokers [3].

Mitochondrial defects have long been proposed to play an important role in the development of cancer. The Warburg effect, an increase in glycolysis that is maintained in conditions of high oxygen tension (“aerobic glycolysis”) and gives rise to enhanced pyruvate and lactate production, is now considered a hallmark of cancer [4], [5]. Recently, studies from Lu and colleagues suggested that glycolytic products, like pyruvate and lactate, lead directly to HIF1α activation; this further boosts metabolism, as well as stimulate angiogenesis and invasiveness, and in turn confers a growth advantage to cells [6]. Interestingly, mitochondrial mutations, occurring with the frequencies ranging from 30% to 70%, contribute to a malignant phenotype via increased ROS, up-regulation of Pyruvate Dehydrogenase Kinase (PDK) 2, attenuating Pyruvate Dehydrogenase (PDH) activity, elevating pyruvate and lactate production, and thereby HIF1α stabilization [7], [8].

To study the mechanism of tobacco smoke mediated HNSCC development, we have previously established a chronic CSE-treated oral keratinocyte model [9]. With this model, in this study, we further characterize the association between tobacco smoke and mitochondrial dysfunction. Here we found that chronic tobacco exposure contributed to cell growth through increased ROS production, upregulation of PDK2 expression, decreasing PDH activity, elevated pyruvate and lactate production, and ultimately HIF1α stabilization. This study provides insight into the functional role of mitochondria in chronic tobacco smoke induced early cellular changes during HNSCC initiation.

## Materials and Methods

### Preparation of Cigarette Smoke Extract (CSE)

CSE was prepared as we previously described according to a modified Carp and Janoff method [9], [10]. Research-grade cigarettes, 2R4F, from the Kentucky Tobacco Research and Development Center at the University of Kentucky were smoked to 0.25 cm above the filter. 100% CSE was prepared by bubbling smoke from one cigarette into 1 ml of PBS. Each puff was 2 seconds long at a rate 35 ml/second. This extract was then filtered using a .22 um filter from BD biosciences filter. Each dilution was done by volume in media. Treatment concentration was .1%. Cells that were grown in a normal incubator that did not have any cell lines treated with CSE are labeled as control-CTRL. The CSE (100%) was stored in sterile Eppendorf tubes at −80 degree Celsius.

### Cell culture and reagents

Immortalized human oral keratinocytes (OKF6/TERT1) were a generous gift from James Rheinwald at Brigham and Women's Hospital in Boston, MA. They retain the normal growth and differentiation characteristics of primary human oral keratinocytes. The cell line was expanded and passaged in keratinocyte serum-free medium (Gibco/Invitrogen; 10725-018). This medium was supplemented with BPE (25 ug/ml), epidermal growth factor (0.2 ng/ml), CaCl_2_(0.4 mM) and 1% penicillin-streptomycin. Both the cells treated with CSE and passaged cells were cultured in 37°C humidified air incubators with 5% CO_2_. All cell lines were grown in 35 mm dishes. The components of Krebs-Henseleit buffer were 5.5 mmol/L glucose, 1.3 mmol/L CaCl_2_, 1.3 mmol/L MgCl_2_, 124 mmol/L NaCl, 3.5 mmol/L KCl, 1.25 mmol/L K_2_HPO_4_, and 26.3 mmol/L NaHCO_3_ (pH 7.5), after bubbling with 5% CO_2_ in air. Where indicated, glucose was replaced by the indicated concentrations of agents.

### Antibodies and reagents

Mouse monoclonal anti-HIF1α antibodies were purchased from BD Biosciences. Rabbit polyclonal anti-PDK2 antibodies were purchased from Abgent. Rabbit polyclonal anti–phosphorylated PDH antibodies were generated from Dr. Verma's laboratory. Mouse monoclonal anti-PDH E1α subunit antibody and 2′,7′-dichlorofluorescein diacetate (DCFH-DA) were from Invitrogen. Dichloroacetate, N-acetylcysteine, and ascorbate were purchased from Sigma. The HIF1α small molecule inhibitor NSC134754 was obtained from National Cancer Institute through their Developmental Therapeutic Program.

### Proliferation assay and Colony Formation Assay

We evaluated the ability of conditioned medium samples to induce cellular proliferation by using MTT assays [7]. The absorbance was measured at 570 nm with a SpectraMax M2e Microplate reader purchased from Molecular Devices (Sunnyvale, CA). Results were expressed as the mean ± SEM of results from replicate wells. The colony formation assay was carried out as described previously. The number of colonies per well with a size greater than 30 cells was counted with the Nikon SMZ1500 microscope [8].

### Western blotting

Cells were lysed in radioimmunoprecipitation assay buffer supplemented with proteinase inhibitor. Whole protein extracts (∼30 µg) were resolved on a 4% to 10% NuPAGE gel (Invitrogen), transferred to a polyvinylidene fluoride membrane (Amersham Biosciences), probed overnight at 4°C with the antibody against PDK2, PDH, p-PDH, HIF1α, and actin, and then revealed using the enhanced chemiluminescence system (Roche). Densitometry was done using Image J software (NIH).

### Measurement of lactate, glucose uptake and ATP levels

Pyruvate and lactate content in the medium was measured by the enzymatic method using a commercially available fluorescence-based assay kit or using the CMA 600 Analyzer (CMA-Microdialysis) as described previously. Cell number was determined using a Coulter particle analyzer [7], [11]. Glucose in the medium were quantitated using the amplex red glucose/glucose oxidase kit (Invitrogen) using a standard curve prepared with serial dilutions of RPMI medium (11 mM glucose) into glucose-free RPMI medium, as described previously [12]. Fluorescence was read using SpectraMax M2e Microplate reader, and results were expressed as pmols/cell. ATP levels were assessed using an ATP bioluminescence assay kit (Roche) [13].

### Real-Time Quantitative Reverse Transcriptase-PCR

Total RNA was isolated with Trizol reagent according to the manufacturer's instructions, and then purified with an RNeasy Kit. One microgram of total RNA was then reverse transcribed into cDNA with Oligo-dT12-18 primers with the SuperScript First-Strand Synthesis kit (Invitrogen). Real-time PCR was performed on ABI PRISM 7000 Sequence Detection System using the QuantiFast SYBR Green PCR kit (Qiagen, Valencia, CA). The primers used for real-time RT-PCR were available upon request. The β-actin was used as an endogenous control for normalization of the expression data of each gene. Each sample was run in triplicate to ensure quantitative accuracy, and the threshold cycle numbers (Ct) were averaged. The results were reported as fold changes (chronic CSE treated cells versus the control untreated cells) and calculated using 2-deltadelta(Ct) method [14], [15].

### ROS measurement

Intracellular ROS generation was assessed using DCFH-DA with the method described previously [16]. Briefly, One million cells per reaction were washed and resuspended in prewarmed (37°C) PBS containing 5 µmol/L CM-H2DCFDA and incubated at 37°C for 15 min to load the dye into the cells. Following the incubation, cells were washed with prewarmed (37°C) growth medium and resuspended in 10 mL growth medium/million cells and incubated at 37°C for 30 min to allow for recovery and conversion of the acetate esters. Cells were washed and resuspended in PBS +1% fetal bovine serum and read by flow cytometry immediately.

### Mitochondrial DNA sequencing

All the DNA samples were sequenced with MitoChip version.2.0, an oligonucleotide microarray, as described previously [8]. Briefly, the entire mtDNA.sequence was amplified in 3 long overlapping PCR fragments, with each reaction containing 50 ng of genomic DNA. The.amplified PCR products then were fragmented and labeled with GeneChip DNA labeling reagent and 30 units/ll terminal deoxynucleotidyl transferase (Affymetrix, Santa Clara, CA). Prehybridization, hybridization, washing and scanning of the MitoChip were performed as described in the Affymetrix CustomSeq Resequencing protocol. All data is MIAME compliant and the raw data has been deposited in a MIAME compliant database (GEO, http://www.ncbi.nlm.nih.gov/geo). The GSE accession number is -GSE24414. Since our MitoChip resequencing data focus only on nucleotide changes, rather than novel DNA sequences, we do not anticipate receiving a GenBank sequence ID at this time.

### Statistical analysis

Differences between experimental variables were estimated using Student's t test as appropriate. A probability level of 0.05 was chosen for statistical significance. The columns in the histograms represent the mean ± SD of at least triplicate values from independent experiments. All computations were done in R 2.11.1 (http://cran.r-project.org/).

## Results

### 1. Chronic CSE treatment induced proliferation in normal oral keratinocytes

**Figure 1:**
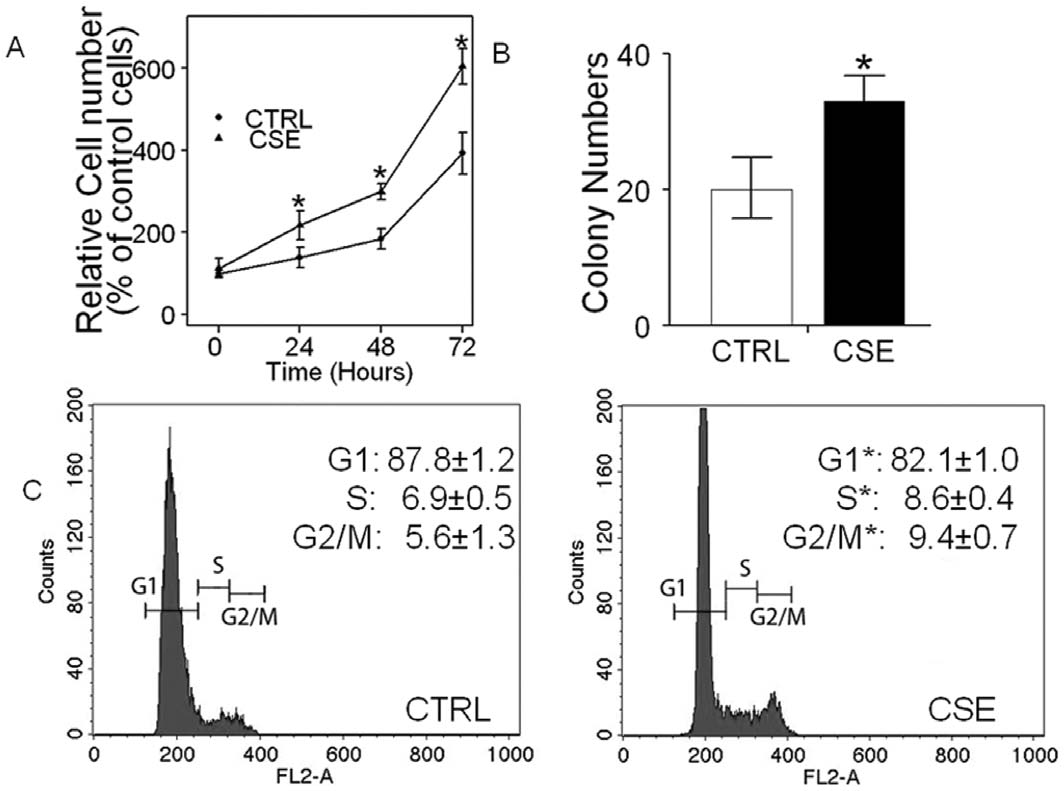
Chronic CSE treatment induced the growth in normal head and neck keratinocyte OKF6 cells. A. In vitro growth of OKF6 cells chronically treated with CSE at the concentration of 0.1% for 7 months, compared with their control untreated cells. The mean absorbance of the control cells at the timepoint of 24 h was set as 100% baseline, and used for data normalization and calculation. B. Colony focus assay showing increased colony formation in the chronic CSE treated cells compared with the control untreated cells. C. Cell cycle analysis of chronic CSE treated OKF6 cells. CTRL, control untreated OKF6 cells; CSE, chronic CSE treated OKF6 cells. Data represent mean±SD of three independent experiments. Student's t test showed significance between the chronic CSE treated OKF6 cells and the control untreated cells (P<0.05).

Previously, we established a chronic tobacco smoke-exposed minimally transformed oral keratinocyte model [9]. In this model, OKF6 oral keratinocyte cells were chronically treated with CSE at the concentration of 0.1% for 7 months. Here, with this chronic CSE treated OKF6 cell model, we further characterize the growth effects induced by tobacco smoke exposure. As shown in Figure 1A, the chronic CSE treated OKF6 cells grew faster than the control untreated cells and at day 3, there was a ∼60% increase in the growth over the control untreated cells (n = 3, P<0.05). We then performed standard colony formation assays on the chronic CSE treated OKF6 cells, and found a significant increase for the chronic CSE treated OKF6 cells when compared with the control untreated cells (P<0.05, Figure 1B). Chronic CSE treated OKF6 cells possessed an accelerated S phase (8.6±0.4% vs 6.9±0.5%, P<0.05) and G2-M phase (9.4±0.7% vs 5.6±1.3%, P<0.05) compared with the control untreated cells (Figure 1C). These data confirm that chronic tobacco smoke exposure induces accelerated growth in normal OKF6 oral keratinocytes.

### 2. Chronic CSE treatment elevated PDK2 expression, increasing PDHα phosphorylation

**Figure 2:**
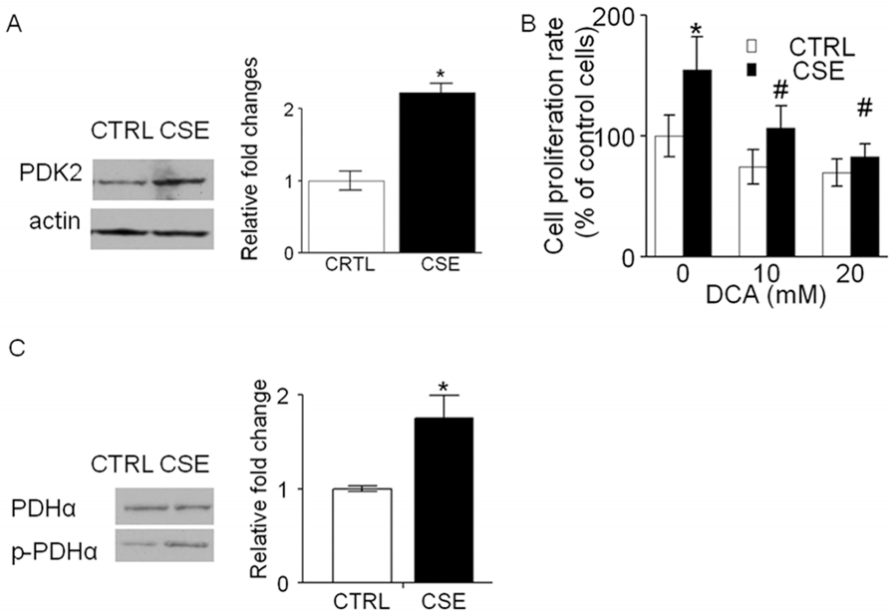
Chronic CSE treatment elevated PDK2 expression, increasing PDHα phosphorylation. A, the protein levels of PDK2 were determined 4 h after switching from culture medium to Krebs-Henseleit buffer in chronic CSE treated OKF6 cells. PDK2 were overexpressed by 2.2±0.1-fold in the CSE treated OKF6 cells versus its corresponding untreated cells (n = 3, P<0.05). B. the growth of chronic CSE treated OKF6 cells were significantly inhibited after treatment with 10 and/or 20 mM dichloroacetate for 48 h. Columns, means of three individual experiments; bars, SD. DCA, dichloroacetate. *P<0.05, #P>0.05. C. western blot for PDHα and phosphorylated PDHα. Results are representative of three independent experiments. CTRL, control untreated OKF6 cells; CSE, chronic CSE treated OKF6 cells.

Recently we reported that inhibition of PDC activity via enhanced expression of PDK2 contributes to the Warburg metabolic and malignant phenotype in human HNSCC; and inhibition of PDK2 lowers the phosphorylation of PDHα and reverts the Warburg metabolic and malignant phenotype [11]. Thus, we examined whether PDK2 contributed to the growth induced by chronic CSE treatment in OKF6 cells. As shown in Figure 2A, elevated expression of PDK2 (2.2±0.1 for PDK2, n = 3, P<0.05) was observed in the chronic CSE treated OKF6 cells compared with the control untreated cells. Further, we observed that the growth was inhibited in chronic CSE treated OKF6 cells after 2 days of treatment with 10 and/or 20 mM dichloroacetate (a PDK2 small molecule inhibitor) to a greater extent than was noted in the control untreated cells (Figure 2B). To determine whether the dysregulation of PDH was triggered by the PDK2 upregulation, we measured the expression of PDH and phosphorylated-PDH (p-PDH) via western blot and determined the ratio of p-PDHα to total PDHα [7]. The ratio of p-PDHα to total PDHα in chronic CSE treated OKF6 cells was found 1.7±0.3-fold higher than that in the control untreated cells (Figure 2C).

### 3. Chronic CSE treatment increased pyruvate and lactate production, accompany by enhanced glucose consumption and ATP levels

**Figure 3:**
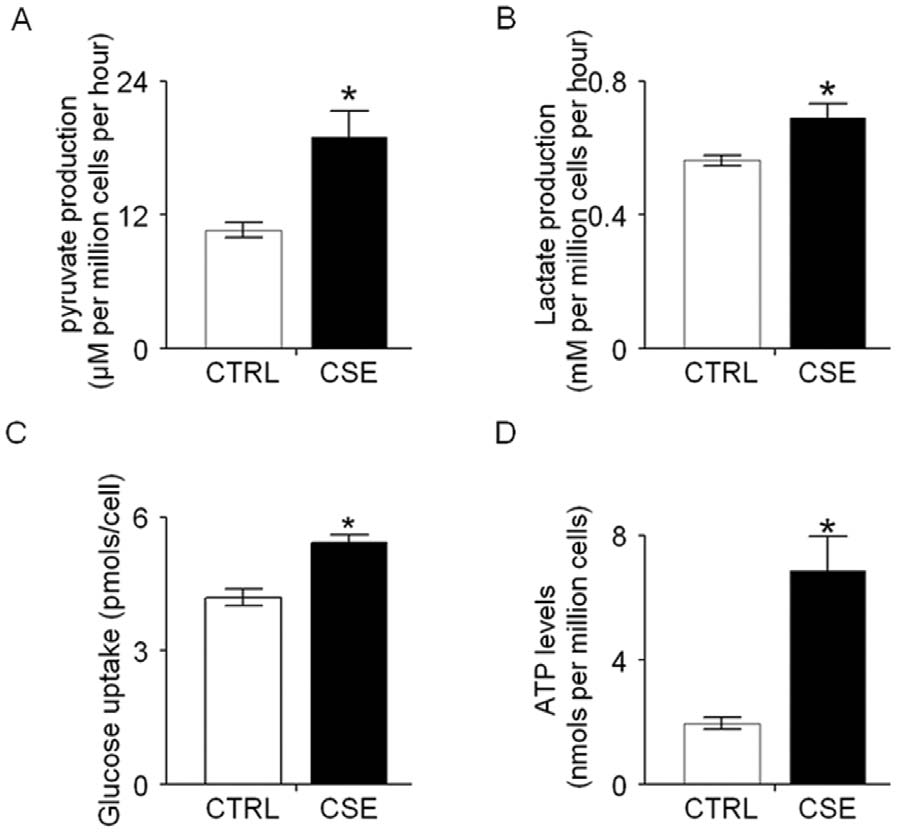
Chronic CSE treatment increased pyruvate and lactate production, accompany by enhanced glucose consumption and ATP levels. A and B, the production of pyruvate and lactate in the culture buffer was measured in chronic CSE treated OKF6 cells. The cells were allowed grown in 35 mm petri dish in 2 ml Krebs media for 6 h, and then the media were collected and assayed for concentrations of pyruvate and lactate. C. the glucose uptake of chronic CSE treated OKF6 cells was significant increased compared with that of the control cells after 48 hour culture in keratinocyte serum-free medium. D. the ATP level in chronic CSE treated OKF6 cells was significantly increased compared with that of the control cells. Triplicate samples were used in each group. *P<0.05. CTRL, control untreated OKF6 cells; CSE, chronic CSE treated OKF6 cells.

Given the elevated PDK2 expression and the increased ratio of p-PDHα to total PDHα, we hypothesized that chronic CSE treatment contributed to the growth of normal head and neck keratinocyte via increased pyruvate and lactate production. As shown in Figure 3A and 3B, we found significantly increased pyruvate and lactate production in chronic CSE treated OKF6 cells compared with the corresponding control untreated cells. To investigate whether this is accompanied by increased glucose uptake and ATP levels, we monitored the glucose uptake and ATP levels in the chronic CSE treated OKF6 cells. As shown in Figure 3C, glucose uptake was significantly higher in the chronic CSE treated OKF6 cells than that in the control untreated cells. Further, we observed that chronic CSE treated cells displayed increased ATP levels in comparison to the control untreated cells (Figure 3D). Together, these data show increased aerobic glycolysis in normal oral keratinocytes exposed to chronic CSE treatment.

### 4. Chronic CSE treatment induces HIF1α accumulation via enhanced lactate production

**Figure 4:**
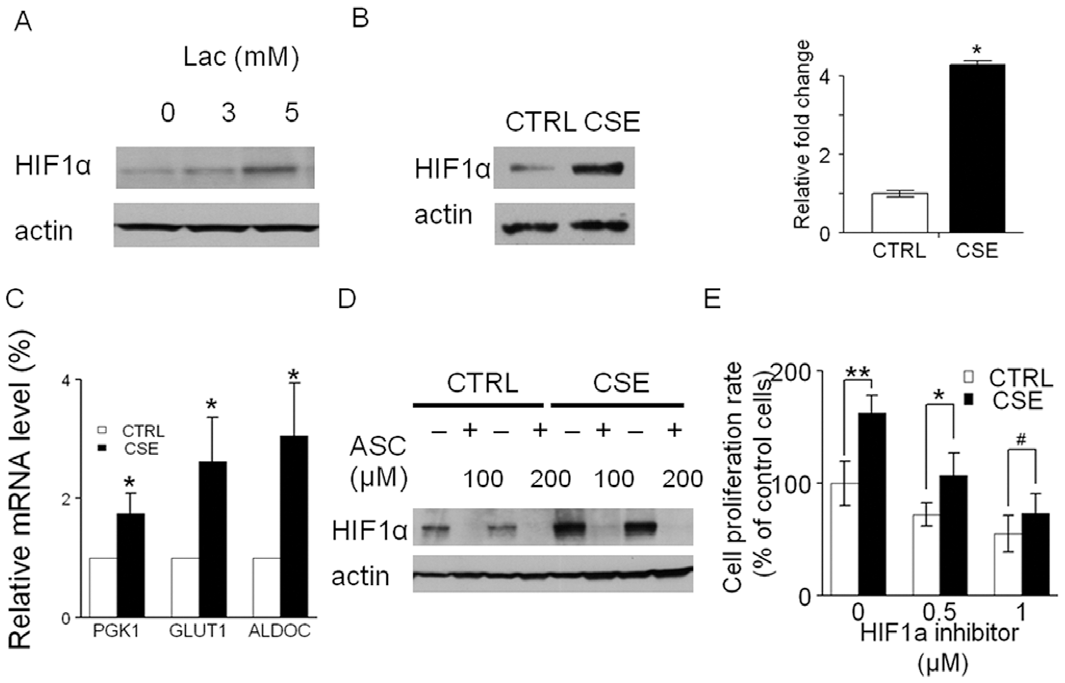
Chronic CSE treatment induced HIF1α accumulation via enhanced lactate production. A. HIF1α levels in OKF6 cells were determined after 4 h of culture in glucose-free Krebs buffer containing 3 or 5 mmol/L lactate. Treatment with 3 and 5 mmol/L lactate caused HIF1α accumulation by 1.3±0.1-fold and 1.9±0.2-fold, respectively. B. the protein levels of HIF1α were determined 4 h after switching from culture medium to Krebs-Henseleit buffer in chronic CSE treated OKF6 cellsHIF1α was overexpressed by 4.4±0.5-fold in the chronic CSE treated OKF6 cells versus their corresponding control untreated cells. C. quantitative RTPCR analysis for three HIF1α-regulated downstream genes, GLUT1, ALDOC and PGK1 in chronic CSE treated OKF6 cells. D. Introduction of ascorbate decreased HIF1α expression in chronic CSE treated OKF6 cells. ASC, ascorbate. E. the growth of chronic CSE treated OKF6 cells were inhibited after treatment with NSC134754, a HIF1α inhibitor. Cell growth was determined by using MTT assay, after exposure for 48 h to 0.5 and 1 µM of NSC134754. Data are mean ± SD values from three independent experiments. **P<0.01, *P<0.05, #P>0.05. CTRL, control untreated OKF6 cells; CSE, chronic CSE treated OKF6 cells.

Cancer-specific aerobic glycolytic metabolites have been shown to promote HIF1α activation under normoxia conditions by interacting with the HIF prolyl hydroxylases (PHD 1–3) [14]. In OKF6 cells, HIF1α accumulation was observed when exogenous pyruvate or lactate was applied (Figure 4A and reference [7]). It is also reported that the expression of HIF1α is an early event in oral carcinogenesis [17]. Here we proposed that the chronic CSE treatment induced HIF1α stabilization through the increased pyruvate and lactate production. As shown in Figure 4B, HIF1α was found 4.4±0.5-fold overexpressed in the chronic CSE treated OKF6 cells versus their corresponding control untreated cells (P<0.05). In addition, three representative HIF1α downstream target genes, including GLUT1, ALDOC and PGK1, were found to be upregulated in the chronic CSE treated OKF6 cells (Figure 4C). It has been previously reported that at low concentrations, ascorbate can selectively reverse the HIF1α accumulation induced by pyruate and lactate production via activation of PHDs. To investigate whether this occurs in the chronic CSE treated cells, we treated the cells with 100 and 200 µM ascorbate for 24 hours. We found that the HIF1α accumulation could be dramatically abolished by ascorbate treatment in comparison to the control untreated OKF6 cells (Figure 4D). To address the contribution of HIF1α in the growth promoting effect induced by chronic CSE treatment, a HIF1α small molecule inhibitor, NSC134754, was applied to the chronic CSE treated OKF6 cells [18]. Treatment with 0.5 and/or 1 µM of NSC134754 significantly inhibited the growth in OKF6 cells with chronic CSE treatment (Figure 4E). Taken together, these data suggest that HIF1α accumulation is a major mechanism of chronic CSE treatment-induced growth in OKF6 cells.

### 5. Chronic CSE treatment increased ROS, increased PDK2 expression, and enhanced HIF1α expression

**Figure 5:**
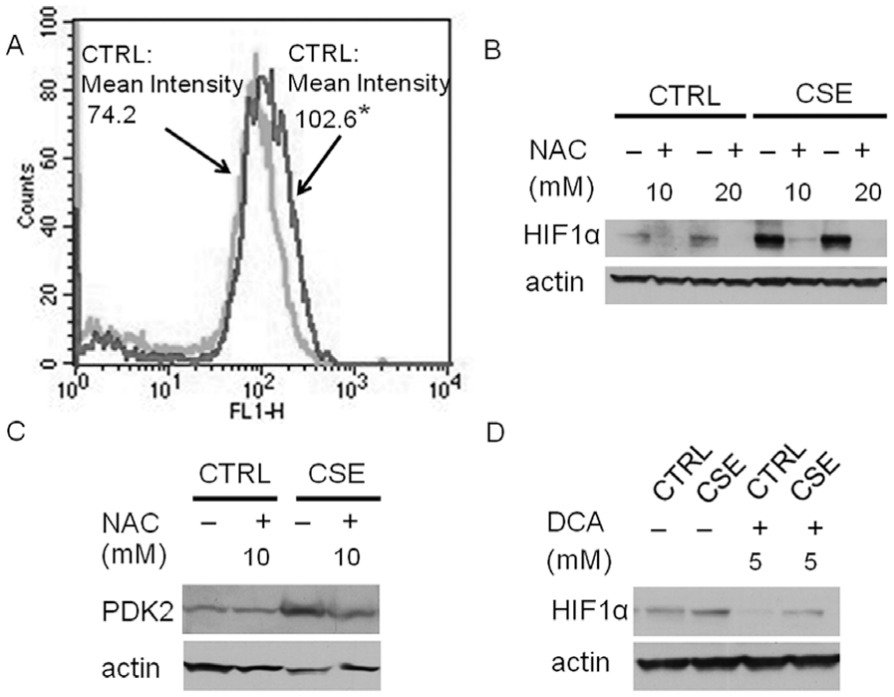
Chronic CSE treatment increased ROS, increasing PDK2 expression, resulting in enhanced HIF1α expression. A, ROS activity was measured in triplicate with the intensity shift from a mean of 74.2 in the control cells (light gray) to a mean of 102.6 in the CSE treated OKF6 cells (dark gary) (n = 3, P<0.05) by flow cytometry. B. In chronic CSE treated OKF6 cells, introduction of ROS scavengers, N-acetylcysteine decreased HIF1α expression. Results are representative of three independent experiments. NAC, N-acetylcysteine. C. In chronic CSE treated OKF6 cells, introduction of ROS scavenger, N-acytylcysteine, decreased PDK2 Expression. NAC, N-acetylcysteine. The experiment was repeated twice with similar results. D. HIF1α levels in chronic CSE treated OKF6 cells exposed to 5 mM dichloroacetate (DCA, PDK2 inhibitor) for 24 h. The experiments were performed twice independently with similar results. CTRL, control untreated OKF6 cells; CSE, chronic CSE treated OKF6 cells.

We hypothesized that chronic CSE treatment induces mitochondrial mutants, thereby initiating increased ROS production, elevated glycolytic metabolism and HIF1α accumulation [7], [8]. To assess this theory, we compared the mitochondrial mutations between CSE treated OKF6 cells and the control OKF6 cells by mitochondrial DNA sequencing. We found 65 nonsysnomoymous amino-changing mutations in CSE treated cells, whereas only 7 nonsynonymous amino-changing mutations in the control OKF6 cell (Table 1, MitoChip sequencing data is deposited in GEO, and the GSE accession number -is GSE24414). Among the genes showing nonsynonymous amino-changing mutaitons in the CSE cells, genes such as ND2, APT6, ND6, COI, and COIII has been linked with ROS production [7], [19], [20]. In consistent, as shown in Figure 5A, we observed ∼40% higher ROS yield in chronic CSE treated OKF6 cells compared to the control untreated cells. To investigate whether the increased ROS generation is responsible for HIF1α accumulation, an ROS scavenger, (N-aceylcystine) was applied to the CSE treated cells. We found that N-acetylcysteine treatment abrogated the HIF1α expression (Figure 5B). Further, we proposed that the increased ROS generation induced by chronic CSE treatment contributes to HIF1α stabilization through the upregulation of PDK2 expression. As shown in Figure 5C, in the chronic CSE treated cells, introduction of N-acetylcystine decreases PDK2 expression. Finally, we found that, after treatment of the chronic CSE treated OKF6 cells with 5 mM dicholoroacetate, a PDK2 small molecule inhibitor, the HIF1α expression level was significantly decreased (Figure 5D).

**Table 1:** Comparison of nonsynonymous amino-acid changing mutations between CSE cells and CTRL cells.

Gene	Total counts of nonsynonymous amino-acid changing mutations	Representative DNA mutations (Position, Amino Acid Alteration)
CSE treated	65	
ND1	8	C->A (3474, T->N); T->G (3665, W->G)
ND2	6	C->G (4825, Q->E); A->T (5095, I->F)
COI	6	A->C (5992, T->P); G->C (6241, A->P)
COII	8	T->C (7633, M->T); G->C (7917, D->H)
ATP6	4	A->T (8735, M->L); T->C (8991, L->P)
COIII	4	T->G (9458, L->R); C->G (9694, L->V)
ND3	3	T->G (10075, L->V); A->C (10315, M->I)
ND4L	2	A->G (10489, I->M); A->G (10637, I->S)
ND4	12	C->G (11434, A->G); T->C (12554, L->P)
ND5	2	C->A (12860, R->S); G->A (12989, A->T)
ND6	2	T->A (14439, Y->F); A->C (14594, I->M)
CYTB	8	G->C (15039, G->R); T->A (15178, V->E)
CRTL cells	7	
ND1	1	A->C (3577, N->T)
COI	2	G->C (6632, Y->F); T->G (7240, Y->D)
ND4L	1	T->G (10685, V->G)
ND5	2	C->G (12986, L->V); G->T (13202, V->C)
CYTB	1	T->C (14806, L->P)

## Discussion

Cigarette smoke is a major risk factor for HNSCC development [21], [22]. In a recent study, we established a chronic CSE treated OKF6 oral keratinocyte as a model for *in vitro* study of tobacco smoke exposure induced HNSCC carcinogenesis [9]. It is postulated that the chronic treatment of cells could best model in vivo cigarette smoke exposure as compared to acute exposure cell line models. With this cell line model, we showed that chronic CSE treatment induced increased cell proliferation rate and anchorage dependent growth, as well as elevated S phase and G2-M phase in oral keratinocytes. We also performed anchorage-independent assays on the chronic CSE treated OKF6 cells, but the results revealed that these cells were not able to form colonies (data not shown). Our data suggests that CSE is capable of increasing proliferation in normal keratinocyte cell lines, and that the growth characteristics represent an early functional molecular change that set the stage for the progression to malignancy.

Recently, interest in the role of PDKs in cancer carcinogenesis has been rekindled. The PDKs control the activity of PDC via phosphorylation of the pyruvate dehydrogenase E1α. In HNSCC, we previously found that inhibition of PDK reverses a metabolic and malignant phenotype [11]. Moreover, PDK2 was found to be overexpressed in HNSCC (unpublished data) as well as other cancers including lung cancer [23]. Bonnet et al. have suggested that PDK2 represents an important target for cancer therapy [24]. In this study, we showed that PDK2 expression is enhanced in the chronic CSE treated OKF6 cells. A further confirmation of PDK2 upregulation is that the ratio of phosphoylated PDHα to total PDHα, which is downstream of PDK2, was increased in chronic CSE treated OKF6 cells in comparison to the control untreated cells. Meanwhile, application of dichloroacetate to the chronic CSE treated OKF6 cells, resulted in inhibition of cell growth [7], [24]. These findings suggested that PDK2 is aberrantly expressed as a result of chronic tobacco exposure, and contributes to the growth-promoting effect induced by tobacco exposure.

Lactate and pyruvate, the end products glycolysis, are overproduced by cancer cells in the presence of oxygen. Given the upregulation of PDK2 and increased ratio of phosphor-PDHα/PDHα, we hypothesized that the Warburg effect is triggered in chronic CSE treated OKF6 cells. We observed increased pyruvate and lactate production, accompanied by enhance glucose uptake and ATP levels, in the chronic CSE treated OKF6 cells, suggesting that the increased glycolysis was induced by chronic tobacco exposure. However, at this point whether the cells use anaerobic or aerobic glycolysis for energy production remains to be determined. The glycolytic metabolites, such as pyruvate and lactate, have been shown to promote HIF-1 normoxic activation by interacting with the HIF prolyl hydroxylases (PHD 1–3) independently [25]. Here we examined whether altered HIF1α expression occurred in the OKF6 cells upon chronic CSE treatment, corresponding to the increased production of pyruvate and lactate. Our data showed that HIF1α expression was significantly upregulated in the chronic CSE treated OKF6 cells. Further, three downstream targets of HIF1α, including GLUT1, ALDOC, and PGK1, showed increased expression in the chronic CSE treated OKF6 cells compared with the control untreated cell. In consistent with Lu et al's study, we showed that ascorbate treatment could abolish the accumulation of HIF1α caused by increased pyruvate and lactate production in the chronic CSE treated OKF6 cells [25]. Notably, use of a HIF1α small molecule blocked the chronic CSE-induced cell growth in OKF6 cells. These suggested that CSE exposure may induce the growth of oral keratinocytes through constitutive activation of HIF1α. In agreement to our data, Zhang et al. reported that nicotine, the major component in cigarette smoke, can stimulate HIF1 protein accumulation and HIF1α contribute to nicotine-promoted cells invasion phenotypes in lung cancer cells [26]. It is also noted that cigarette smoke exposure impairs angiogenesis by inhibiting VEGF through decreased expression of HIF-1alpha in hypoxic condition [27]. Moreover, Lin et al. found that the mean nuclear HIF1α labeling indices increased significantly from normal oral mucosa, through mild-, modertate-, and severe- epithelial dysplasia to oral squamous cell carcinoma, suggesting HIF1α is an early event in oral carcinogenesis [17]. Consistent with this, positive HIF-1α immunostaining was also found in the parabasal to middle third layer of the benign cervical squamous epithelium and HIF-1α expression significantly increases in all grades of dysplasia [28].

Interestingly, in our chronic CSE treated OKF6 cell model, we have found that more amino acid changing mitochondrial mutations than the control untreated cells. Our data supported that the chronic CSE treatment may induce ROS production through mitochondrial mutation, which in turn upregulated PDK2 and HIF1α expression. First, increased ROS production was observed in the chronic CSE treated OKF6 cells in comparison to that in control cells. Second, inhibition of ROS production by ROS scavenger N-acetylcysteine blocked PDK2 expression and HIF1α expression in chronic CSE treated OKF6 cells [19], [29]. It is reported that N-acetylcysteine has been used as an anticancer agent in preclinical models, and N-acetylcysteine treatment of the cybrids carrying ROS-generating mitochondrial mutants dramatically reduced the amount of ROS production [19]. Third, inhibition of PDK2 expression with dichloroacetate abrogated the HIF1α expression. It should be of note that no experimental proof is provided that mitochondrial mutations found in the paper are responsible for the altered growth rates, ROS production, or HIF1 accumulation, and therefore additional studies regarding this point are warranted.

In summary, the present study revealed the growth-promoting effects induced by chronic tobacco exposure as early cellular changes that lead to malignancy. As showed in Figure S1, we proposed that, in human normal oral keratinocytes, tobacco exposure induced mitochondrial mutations and increased ROS productions, thereby sequentially facilitating upregualtion of PDK2, attenuation of PDH activity, and subsequent increased aerobic glycolytic activity (increased pyruvate and lactate production), resulting in the HIF1α accumulation and the growth-promoting transforming phenotype. Using the chronic CSE-treated human oral normal keratinocyte model, the present study demonstrates a role of chronic tobacco exposure in the induction of aerobic glycolytis and HIF1α stabilization during HNSCC initiation. Although the use of immortalized keratinocytes in the model to study the chronic effect of CSE has some limitations as these cells as not normal, we are confident that this study will provide potentially important insights into mechanism of human HNSCC carcinogenesis by chronic tobacco smoke.

## Supporting Information

Figure S1 **The proposed mechanism on chronic CSE induced HNSCC initiation.**
(TIF)
