# Supplementary figures and images for: Chronic CSE Treatment Induces the Growth of Normal Oral Keratinocytes via PDK2 Upregulation, Increased Glycolysis and HIF1α Stabilization
Source: PLoS One. 2011 Jan 19;6(1):e16207. doi: 10.1371/journal.pone.0016207 (PMC3023770; doi:10.1371/journal.pone.0016207)

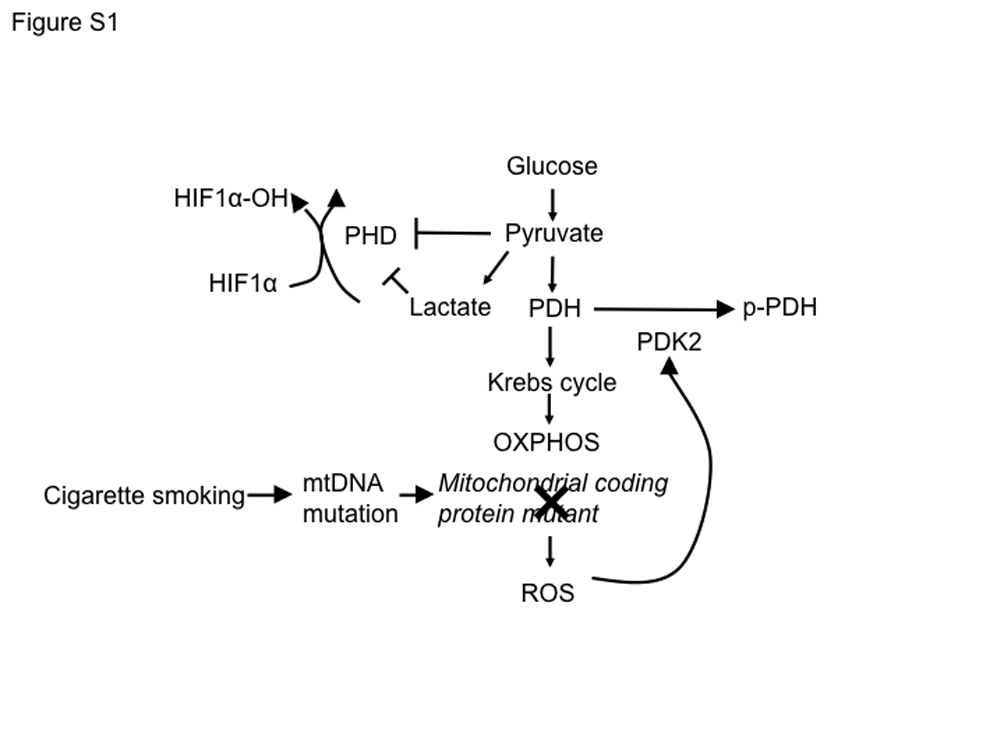

Supplement: Figure S1 — The proposed mechanism on chronic CSE induced HNSCC initiation. (TIF) [file pone.0016207.s001.tif]
